# The Application of RBF Neural Network Model Based on Deep Learning for Flower Pattern Design in Art Teaching

**DOI:** 10.1155/2022/4206857

**Published:** 2022-06-13

**Authors:** Lijun Xiao, Yan Luo

**Affiliations:** ^1^School of Fine Arts and Design, Changsha Normal University, Changsha, Hunan, China; ^2^School of Economics and Trade, Hunan University, Changsha, Hunan, China

## Abstract

The rapid growth of artificial intelligence technology has been deployed in art teaching and learning. Radial basis function (RBF) networks have a completely different design compared to most neural network architectures. Most neural networks consist of multiple layers that can introduce nonlinearity by repetitive application of nonlinear activation functions. In this research, people will study the application of the RBF neural network model based on deep learning in flower pattern design in art teaching. The image classification process is finding and labeling groups of pixels or vectors inside an image based on rules. Deep learning is a type of machine learning that uses artificial neural networks to replicate the structure and function of the human brain. The proposed model uses the RBF neural network-based deep learning model in flower pattern design in art teaching and provides efficient results.

## 1. Introduction

In recent years, there has been a growth in the number of websites dedicated to Chinese painting and art in general. Because of the simplicity with which they may be preserved and made accessible, digitised Chinese paintings are becoming increasingly desirable as historical artefacts [1]. Because of the ease with which these Xuan paper artworks can be digitised, they can be quickly transformed into digital paintings as well. As a result, digitising Chinese art has become more essential as a conservation approach in the country. For specialists, categorizing unsigned digital Chinese artworks of various types is challenging due to their inability to quickly identify the most relevant category [2]. As a result, there has been a rise in interest in digital Chinese painting authenticity-assisted verification of categorization accuracy and automation and in automated Chinese painting authentication. Because of the digitisation of traditional Chinese artworks, it is becoming increasingly difficult to verify their authenticity [3]. There has never been a time in history when the accuracy of digital Chinese artworks was more crucial than it is today in the age of big data. Traditional techniques of identification rely substantially on the expertise of specialists in the field of identification to be successful. A substantial challenge to current authentication approaches is posed by modern counterfeiting of information technology because of the lack of objectively referencing signals and the inherent subjectivity of such procedures [4]. The authentication of the artworks necessitates the use of digital Chinese artwork identification technology that is both automatic and exact. At the beginning of the twentieth century, Western art experienced an unparalleled crisis, which resulted in the establishment of a number of art schools [5].

The avant-garde movements of Cubism, Futurism, and Dada are mainly responsible for the birth of interactive art in the form that people are familiar with today [6]. Since its inception in 1956, the term “artificial intelligence” has been associated with interactive art in some way or another. Since the beginning of time, artificial intelligence (AI) has been included in interactive art in many forms [7]. At a time when interactive art was still in its infancy, aesthetics and technological execution took precedence over the user experience and cognitive style of operation. Respect must be dealt with in a more nuanced manner, considering the user's prior experience and their current cognitive state [8]. The development of voice recognition and computer vision in the 1980s marked the beginning of AI research in the United States. Because of AI, new technologies and concepts have been developed that enable modern digital interactive art, have an influence on public aesthetics, and have an impact on the creative thinking of artists (AI) [9]. In the future, the creative interplay between artistic expression and technological progress will be a driving force behind breakthroughs in art and human-computer interaction. AI has a profound impact on human cognition and on how we perceive and create art [10]. As a result, for our dissertation topic, people are collecting data on AI, movie and television production, administration, and consumer viewing experiences. It includes a diverse range of materials, including anything from books and research articles to films and television episodes [11]. In order to have a deeper understanding of the problem, the research team conducts both macro- and microinvestigations. Through the use of deconstruction and reconstruction thinking, this study investigates the impact of AI on interactive creative expression in terms of both technology and thinking [12]. In this new interactive art concept, AI has deconstructed the old interactive art concept that was based on time, space, and natural logic and rebuilt it in a different way.

The efficiency of intelligence classification is partly contingent on the categorization technique used and the way in which characteristics are handled [13]. Monte Carlo convex models may be used to sort a large number of subcategories of Chinese art. In order to fully automate the statistical model-based classification procedures discussed above, the time-consuming process of modifying parameters must be undertaken. Once the wavelet properties of the artwork are acquired by the support vector machines, they are employed [14]. Despite advancements in parameter adjustment, the precision of feature quantification was not high enough. Using deep learning-based classification approaches, it is possible to offer pictures with high-level semantic representations while also increasing the automation of categorization. With the use of recurrent neural networks, people were able to create a painterly classification of Picasso's brushstrokes (RNNs) [15]. To categorize and interpret ancient works of art, deep convolutional neural networks are used in conjunction with machine learning techniques (DCNNs). The brushstroke features of ink paintings can be quantified using CNN, bringing the categorization of the style to a close [16]. CNN's ability to recognize specific patterns and properties is improved by using a deep aggregation structure. The use of residual processes and jump connections to increase the depth of the network can improve classification performance by increasing the network's depth [17]. The understanding of fractal theory among graphic artists is very inadequate. The aesthetic of fractals has become increasingly popular in science, while the art world has remained mainly uninterested in the subject [18]. A cursory understanding and judgement of fractal art is stressed, rather than digging further into its theory and use in design, as is the case in this article. As a result, it is clear why this phenomenon has manifested itself in the first place. Take a look at how fractal art is created with today's fast-expanding computer and software technology [19]. The first stage in every kind of creative expression is to ensure that it is functional, and then comes the aesthetics. Cultural influences have emerged as a means of promoting uniqueness and regional differences in the field of fractal art. When creating new fractal art designs, this is an important factor to keep in mind. In part, the competitive advantage of fractal art may be attributed to the individual characteristics of its creators. If designers and design organisations want to create localised fractal art patterns that are based on the characteristics of the original fractal art patterns, they must first address this challenge, which is a critical one to solve [20]. This method uses RBF networks to produce and analyse fractal art patterns, emphasising the culture-related function of the patterns through the application of connotation and quality in the patterns, in addition to the measures and processes that are employed in the production of culture. This strategy may be valuable to designers in certain situations [21]. This article investigates the process of creating fractal art patterns and then generates and evaluates fractal art patterns that embody the essence of cultural identity as a whole. With the passage of time, the patterns in fractal art that have been present for a long time will become more regular and repeatable. Fractal art patterns are influenced by the RBF network approach used in data processing and development [22].

Fractals are pictures in art that are tumultuous and uneven. Many portions of the artwork use irregular geometric forms and disconnected visual structures to convey their message, which is seen in many of the pieces. Our cosmos is composed of fractal art pictures that follow a set of laws that guide its creation [23]. It is necessary to analyse and remove data inconsistencies caused by individual data changes in order to make fractal art designs, and this is accomplished via the use of colour analysis technology and the RBF network technique. This method eliminates the possibility of human bias having an impact on the final results. When using an RBF network technique, nodes are employed to process superpixel blocks in order to enhance colour space, reduce background area, and ensure accuracy. The use of an RBF network can significantly reduce the number of times an algorithm must be run in a given situation [24]. Despite the fact that there are hundreds of millions of variations in the fractal painting, the patterns stay constant. They are yearning for a sense of security in the midst of constant change and uncertainty. Fractal art photos have a lot of aesthetic and structural traits in common with each other [25]. Fractal graphics, on the other hand, can include an infinite number of layers, but the final shape is unexpected. In part due to the usage of fractal geometry, the score art images have a strong feeling of depth to them. Even though fractal pictures are continually changing, they never lose their feeling of balance. When it comes to aesthetic appeal, dynamic and harmonic visuals outperform static graphics hands down. This study focused on the application of the RBF neural network model based on deep learning in flower pattern design in art teaching.

### 1.1. Motivation of the Study

The low-quality floral pattern in art images is highly challenging to teach image restoration in affirmative first-order flower pattern design associated with and without off-digital holographic objects. This article addresses the mentioned challenges and proposes a method for recognizing the initial floral pattern design in an art teaching image of an RBF algorithm. The variables of such lighting illumination are studied, and the maximum average standard errors with conventional items are 7.6 percent. The gigantic space weaving technique in coordinates is chosen in digital parabolic geometry technology to overcome these splicing variables. RBF algorithm is employed to convert these nonlinear equations into optimized ones. Finally, the utilisation of the technology for the 3D display of such a traditional revolving three-dimensional art mechanical structure is achieved using high-resolution weaving.

## 2. Materials and Methods

**Figure 1:**
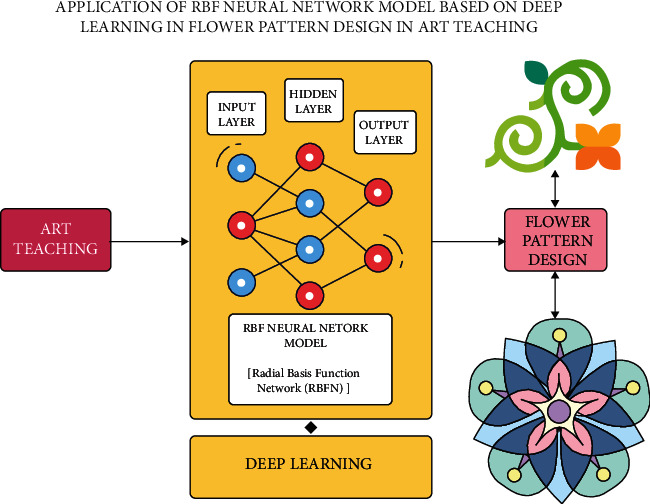
Architectural diagram.

The proposed model makes use of radial basis function (RBF) neural networks primarily based on the deep learning model in flower pattern design in art teaching. RBF networks are unique in that they have a completely different design compared to most neural network topologies. In most neural networks with several layers, nonlinearity is produced by repeatedly applying nonlinear activation functions. The RBF network, on the other hand, is made up of only three layers: an input layer, a hidden layer, and an output layer (refer Figure 1).

The input layer is not a process layer; it just receives data and feeds it into the RBF network's hidden layer. The mechanism that occurs at the hidden layer differs significantly from that of other neural networks, and this is frequently where the RBF network's power comes from. The output layer is in charge of prediction tasks such as classification and regression. RBF networks are a typical type of artificial neural network used to perform approximation concerns. RBF networks are distinguished from other neural networks by their universal approximation and faster learning rate. The RBF neural network is a type of feed-forward neural network made up of three layers: the input layer, the hidden layer, and the output layer. Each tier is responsible for its own set of tasks. Deep learning is a type of machine learning-assisted artificial neural network in which multiple layers of processing are trained to extract higher and higher-level alternatives from data. Supervised, semisupervised, and unsupervised learning are common.

**Figure 2:**
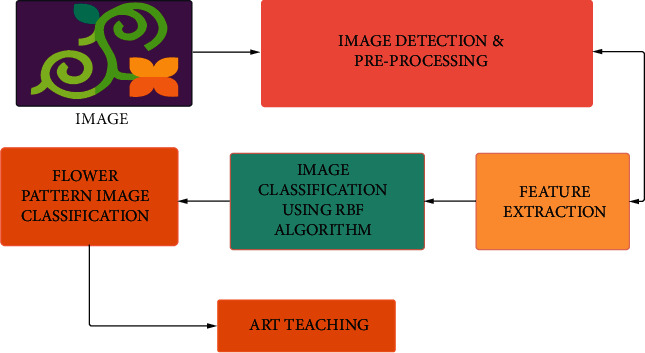
Block diagram on RBF algorithm doing image classification.

In multimedia processing, image classification is a fascinating problem. Many researchers have recently introduced RBF-based algorithms for picture categorization. Traditional RBF neural networks, on the other hand, were sensitive to centre initialization. It must locate relevant features for further RBF clustering to acquire acceptable centres. Furthermore, the standard RBF training approach is time-consuming. The art teaching process is carried out with the help of image classification. The practice of identifying and labeling groups of pixels or vectors inside an image based on certain rules is known as image classification. One or more spectral or textural properties can be used to create the classification law. There are two types of classification methods: supervised and unsupervised. The image classification process is shown in Figure 2). Image inputs are given to the image detection and preprocessing mode; then the data are sent to the feature extraction. After feature extraction, it is sent to the RBF-based algorithms for picture categorization. This technique is used in our proposed model to classify images of the flower design, which is incorporated into a teaching program. Along with the use of machine learning and deep learning, AI is becoming a normal element of the technology landscape. Deep learning is employed in the art teaching field and has been found to deliver excellent results.

### 2.1. RBF Neural Network Algorithm Based on Deep Learning

In Cartesian coordinates, the quantitative idea for digital high resolution of The Museum of Modern Art Collection: Distinct Southern Cross Flight is *p*=0. *K* is described as a recreated aircraft that is important when compared to charge-coupled devices (CCD) display flight with a distance of *p*=*g*_1 _ and secant to the surface of such a specimen's surface.

The nonoptically simplified geographic exterior is highlighted to light signal according to the quantitative advanced optical construct, and the diffracted frame spectrum of such an organism's surface *p*=0  could also be regarded as the scattered light of a large number of diffraction pattern representations, whereby the quantum superposition of all of the simplistic diffraction pattern waves can be calculated using the RBF algorithm with lightweight deep learning.

(1) $$\begin{array}{c} K=\sum_{j=1}^{x}j\vartheta_{x}-\text{jdp}+\sum_{S\in T}^{n>m}S_{T}k\left(n,k,p\right)\times \sum_{n=1}^{p}j\vartheta \left(n,k,p\right). \end{array}$$

The frequencies of dispersion specular at comparable spots alone on the surfaces of 3D art in equation |*S*_*T*_*k*|^2^ denote the frequencies of dispersion specular  *ϑ*_*x*_  at comparable spots alone on the surfaces of 3D art in (1). Because the density of an object was not of primary interest here, −*π* :  *π* denotes the frequencies of dispersion specular at comparable spots alone on the surfaces of 3D art. Lighting spectrums are denoted as $j=\sqrt{-1}\text{ and }k=2\pi /\gamma ,\;\gamma .$

The method can be proved in (2) if the illumination orientation is comparable to *nkp* and the angle between the brightness and the axis *p* is

(2) $$\begin{array}{c} \vartheta =\sum_{S\in T}^{n>k}S_{T}k\left(n,k,p\right)+\int_{n}^{\rho }\frac{2\pi }{\gamma }\left(n\;\sin\;\rho -p\;\cos\;\rho \right)+\sum_{j=1}^{x}j\vartheta_{x}-\text{jdp}. \end{array}$$

The illumination irradiation angle representation is provided using (3). The final process evaluation shows that if one wavelength is used for emphasis, the forecast angle may be changed to detect distinct reproduced fields, which can then be transferred to form contours, allowing for multiart creation evaluation using RBF neural network algorithm based on deep learning.

(3) $$\begin{array}{c} \vartheta_{1}\left(n,k\right)=\sum_{j=1}^{x}j\vartheta_{x}-\text{jdp}+\sum_{n=1}^{\rho }\frac{2\pi }{\gamma }\left[n\;\sin\;\rho -p\left(1+\cos\;\rho \right)\right]+\vartheta_{x1}. \end{array}$$

The process possibilities of a source of light *ϑ* *an*  *d* *ϑ*+∇*ϑ* in the reproduced optical viewfinder are, together with

(4) $$\begin{array}{c} \gamma =\int_{\rho }^{n=1}\frac{2\pi }{\gamma }\left\{n\left[\sin\;\rho -\left(\rho +\nabla \rho \right)\right]-\overset{\rho }{\underset{p=1}{\sum }}p\left[\cos\;\rho -\cos\left(\rho +\nabla \rho \right)\right]\right\}. \end{array}$$

Whenever a magnetic source with wavelength portrays an item utilising parallel illumination with an inclination angle of illuminating light,

(5) $$\begin{array}{c} \vartheta_{1}\left(n,k\right)=\int_{\rho }^{n=1}\frac{2\pi }{\gamma }\left\{n\left[\sin\;\rho -\left(\rho +\nabla \rho \right)\right]-\overset{\rho }{\underset{p=1}{\sum }}p\left[\cos\;\rho -\cos\left(\rho +\nabla \rho \right)\right]\right\}+\sum_{x=1}^{x=2}\vartheta_{x1}\;-\vartheta_{x2}. \end{array}$$

Each *ϑ*_*x*1_, *ϑ*_*x*2_ in (4) with (5) represents a   − *π* :  *π*,   with uniform chance sampling procedure. The following equation represents the simplified version of the above two equations:

(6) $$\begin{array}{c} \vartheta_{2}\left(n,k\right)=\sum_{x=1}^{x=2}\vartheta_{x1}-\vartheta_{x2}+\int_{n=1}^{k}\frac{2\pi }{\gamma }\left\{n\;\sin\left(\rho +\nabla \rho \right)-\sum_{p=1}^{\rho }p\left[1+\cos\left(\rho +\nabla \rho \right)\right]\right\}+\vartheta_{x2}. \end{array}$$

However, the difference is tiny, the same light beam has merely a 3D art effect, and also the diffracted qualities of the same waveguide on the surface of an item do not need to change significantly. *ϑ*_*x*1_, *ϑ*_*x*2_ is no longer a random occurrence of   − *π* :  *π* and a unified value is now possible. Because the RBF algorithm technique has been applied to relatively tiny datasets, cos  ∇*ρ* ≈ 1, sin  ∇*ρ* ≈ ∇*ρ* and  *ϑ*_*x*1_, *ϑ*_*x*2_=*ϵ*2*π*(0 < *ϵ* < 1) that appear to be phase measurement noise types may be recast, as shown in

(7) $$\begin{array}{c} \nabla \rho \left(n,k\right)=\sum_{p=1}^{\rho }p\left[1+\cos\left(\rho +\nabla \rho \right)\right]+\sum_{n=1}^{\rho }\frac{2\pi }{\gamma }\left(-n\nabla \rho \;\cos\;\rho -\sum_{p=1}^{\rho }p\nabla \rho \;\sin\;\rho \right)+\epsilon 2\pi . \end{array}$$

The Laplace transform is made up of several components, as shown in (6). These elements are inextricably linked to the object's surface. The distinction has to do with the locational vector *x*, which is also known as the longitudinal tilt term. Changes in its coordinates correspond to a shift in its value. Its operations have more significant effects than those induced by the organism's orbital elevation change, which also results in the phase difference encased *s*. Before process unpacking, remove the linear tilt word from the floral design in the art instruction image to get the variable phase component. The following equation is obtained after background subtraction:

(8) $$\begin{array}{c} p\left(n,k\;\right)\approx \sum_{p=1}^{\rho }p\left[1+\cos\left(\rho +\nabla \rho \right)\right]-\frac{\nabla \rho \left(n,k\right)}{2\pi }\sum_{n=1}^{\rho }\forall_{\rho }-n\;\cos\;\rho , \end{array}$$

where the height of such an object's surface changes with its exact position, which can be characterized as follows:

(9) $$\begin{array}{c} \forall_{\rho }=\sum_{p=1}^{\rho }\left.p\nabla \rho \;\sin\;\rho \right)+\epsilon 2\pi \;+\frac{\gamma }{\nabla \rho \;\sin\;\rho }, \end{array}$$

where ∀_*ρ*_  denotes the difference in object height corresponding to a phasing shift transformation of 2*π*, where it can be observed that this is inversely proportional to the apparatus. The ∀_*ρ*_  preparatory design may be covered if it is higher or equivalent to the overall depth evaluated on this surface using RBF neural network algorithm based on lightweight deep learning.

There is still a longitude and latitude system *Knmp* with and in electronic high-resolution data acquisition system, the *p*=0  surface in which its scattering is located, its own distance of a flower pattern in art trying to teach picture space again after performance capture in 3D art substratum is *p*=0, and the digital flower pattern architecture in better teacher images acquired by CCD entering data is *F*(*n*, *k*); after which, the 1-FFT restoration illumination signal ground might be illustrated by Projector Scat as in the following:

(10) $$\begin{array}{c} H\left(n,k\right)=\ln\left[\frac{j\;d}{2p_{0}}\left(n_{i}^{2}+k_{i}^{2}\right)\right]\times \int_{-\infty }^{+\infty }\left\{F\left(n,k\right)\ln\left[\frac{j\;d}{2p_{0}}\left(n^{2}+k^{2}\right)\right]\right\}.\;\ln\left[-2j\pi \left(\frac{n_{i}}{\gamma p_{0}}n+\frac{k_{i}}{\gamma p_{0}}k\right)\right]. \end{array}$$

The *γ* frequency of such a beam of light and also the frequency of such a transverse stream *k*=2*π*/*γ* are equal in (11). Inside an art instruction image, a reconstructed circular signal with such a distance away of *p*_*s* _ is used to show fused flower design processes.

(11) $$\begin{array}{c} T_{s}\left(n,k\right)=F\left(n,k\right)\ln\left[\frac{j\;d}{2p_{0}}\left(n^{2}+k^{2}\right)\right]+\left[\frac{j\;d}{2p_{s}}\left(n^{2}+k^{2}\right)\right]\;. \end{array}$$

The electromagnetic path of propagation of such a beam of light along the range *p*_*i*_ of an undisturbed digital hologram is as follows the :

(12) $$\begin{array}{c} H_{i}\left(n,k\right)=\sum_{n=1}^{k}{\text{FT}}^{-1}\left\{\text{FT}\left[v\left(n,k\right)H^{*}\left(n,k\right)T_{s}\left(n,k\right)\right].\sum^{}\ln\left[{\text{jdp}}_{i}\sqrt{1-\gamma^{2}\left(q_{n}^{2}+q_{k}^{2}\right)}\right]\right\}. \end{array}$$

Equation (13) denotes the hologram's display function, *H*(*x*,  *y*) denotes the item profession in the *p*=0 plane corresponding to a municipal picture of a particular component also on information flower patterns in art teaching image, and *q*_*n*_, *q*_*k*_ denotes the frequency precise location corresponding to (*n*,  *k*).

(13) $$\begin{array}{c} \rho =\sum \ln\left[{\text{jdp}}_{i}\sqrt{1-\gamma^{2}\left(q_{n}^{2}+q_{k}^{2}\right)}\right]\geq H_{i}\left(n,k\right). \end{array}$$

The *Kρϑn* coordinate is related to the rectangular frame. Assume that what a storing placement A appears to have the good characterizations *Kρϑn* in a rectangular point of reference and also the excellent value *A*(*ρ*, *ϑ*, *n*)  in a circumferential reference frame, and the angular displacement between such estimates of a righteous line from input image through axis *N* on the *mkp*  substrates but also the *P* − axis is presented in

(14) $$\begin{array}{c} \rho =\sum_{n=1}^{k}{\text{FT}}^{-1}\left\{\tan\;\vartheta =\frac{p}{m}\geq \rho =\sqrt{m^{2}+p^{2\;}}\right... \end{array}$$

To start looking at this RBF neural network algorithm based on lightweight deep learning, numerous curved surfaces with varying circular centres are projected onto such surfaces, where *n* is the same as in this ordinary 3D art frame. Thefollowing equation can be used to express the distance between both the centres of concentric circles:

(15) $$\begin{array}{c} \rho_{0}^{\prime }=\sqrt{{\left(p_{0}\sin\;\vartheta_{0}+\frac{\cos\;\alpha }{\cos\;\beta }n_{1}\right)}^{2}}+{\left(p_{0}\cos\;\vartheta_{0}+\frac{\cos\;\gamma }{\cos\;\beta }n_{1}\right)}^{2}. \end{array}$$

Thefollowing equation shows how numerous circles' facilities are directed in different directions:

(16) $$\begin{array}{c} \vartheta_{0}^{\prime }=p_{0}\sin\;\vartheta_{0}+\frac{\cos\;\alpha }{\cos\;\beta }n_{1}\geq p_{0}\cos\;\vartheta_{0}+\frac{\cos\;\gamma }{\cos\;\beta }n_{1}, \end{array}$$

*p*_0_ and *ϑ*_0_ indicate the distance and the direction of the round 3D art cylinders possibly on the surface of *n*_1_=0, respectively, and the centres of overlapping circles are generated by intersections of the separate cylinders or on the surface.

## 3. Result and Discussion

**Figure 3:**
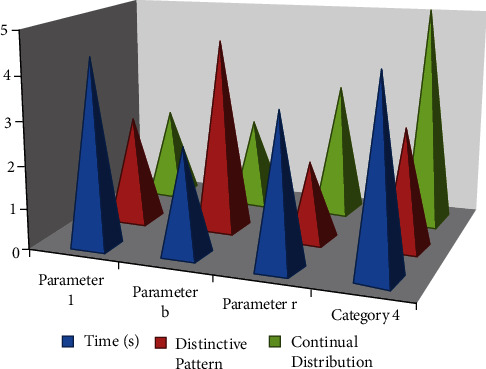
The impact of new flower pattern and lightweight deep learning on traditional flower pattern arts and crafts.

Online educational prerecorded video dataset is considered for training and testing the processes. For transform and nonlinear modifications, a single iterative primitive approach is employed, and in practice, integration of self-designed and secondary translation and rotation are used. The ∀_*ρ*_ − *n*  cos  *ρ*  c-occurrence matrix is used to prepare the image for different modifications before selecting the best image. Each *p*(*n*, *k* ) variable in a dynamic system alters a graph's distinctive properties, such as its form (in Eq. 8). By giving distinct weight coefficients ∑_*p*=1_^*ρ*^*p*[1+cos(*ρ*+∇*ρ*)] to a dynamical emotional bond attribute  ∇*ρ*(*n*, *k*), the transition effect is magnified even further (Figure 3).

Image categorization is a fascinating subject in multimedia processing. Many academics have recently created RBF-based image categorization systems. On the other hand, conventional RBF neural networks were sensitive to initialization in the centre. To get appropriate centres, they must discover significant features for subsequent RBF clustering. Furthermore, the traditional RBF training method is time-consuming. Image classification is used to aid in the art instruction process. After that, the RBF neural network algorithm based on a lightweight deep learning loss optimization technique is employed to increase image quality while lowering noise. The image extracted and purified features from mentoring are compared multiple times to ignore the situation of extremely tilted images due to incorrect weights, summarize the appropriate style charge interval, and finally modify the factors to deliver multiple types of fractal images instantly, attempting to break its single and flower pattern art and crafts form and realizing the additional growth of spatial frequency visual art.

The distinguishable pattern characteristics in terms of quality of parametric a and *b* are both larger than 2, as given in Table 1, meaning that the multitexture is now simple and coherent and hence acceptable for digital picture processing. After filtering out the photographs that are acceptable for merging, the splicing technique is carried out. They are both distributed repeats and continuous cutting, as seen in Figure 3. This type of visual can be spliced by random locations in the parameter as in Table 1, which requires distinct pattern characteristics and regular distribution, and can also be dispersed freely to achieve self-affine, infinite fineness, and independent pattern characteristics of spectral graphics. Figure performance is spliced indefinitely.

**Figure 4:**
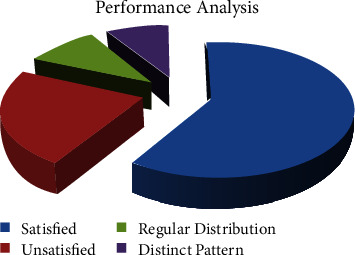
Statistics for modern new flower pattern art and crafts in the performance monitoring of different online medication teaching systems.

The recognizable pattern: the quality characteristics for parametric a and *b* are also both greater than 2, indicating that such multitexture has now become simple but coherent and hence suitable for digital image processing. The merging technique is used after screening out other photographs that seem to be suitable for merging. Both are scattered repeats with continuous cutting. This form of visual can also be spliced by random places in parameter, which demands different pattern characteristics along with regular distribution, and it can also be freely disseminated to produce self-affine, limitless fineness, with independent pattern features of spectral graphics. Figure performance is indefinitely spliced. And traditional deep learning (*n*_*i*_^2^+*k*_*i*_^2^) has increased students' *F*(*n*, *k*) learning performance and passion to some level; it does not fully comprehend students' learning and, as a result, can vary for each individual, and it cannot ensure that every student engages in teaching. −2*jπ*(*n*_*i*_/*γp*_0_*n*+*k*_*i*_/*γp*_0_*k*) will be the (*n*^2^+*k*^2^) technique's incarnation. If humans truly desire educators to be much more actively engaged in the teaching process, along with gathering students' educational experiences and providing new reference direction through private contact, we must focus solely just on the significant improvement discussed in the preceding section, as shown in Figure 4.

**Figure 5:**
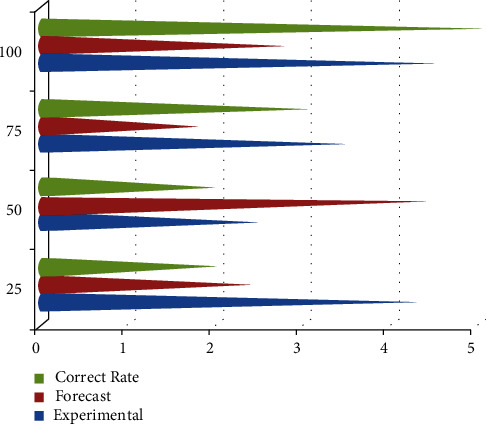
Experimental art and design accuracy analysis of forecast using RBF neural network algorithm.

Image classification is the process of finding and labeling groups of pixels or vectors inside an image based on specific rules. The categorization law can be created using one or more spectral or textural properties. Classification methods are classified into two types: supervised and unsupervised. The image classification procedure is as follows: the image detection and preprocessing mode is used to process image inputs before sending the data to the feature extraction mode. After feature extraction, the image is classified using RBF-based algorithms. The most widely used digital elements in the present education are recording equipment and video screens; however, similar equipment can also be used in home art instruction. Photographs, videos, and other flower patterns could be used by teachers to produce and bring art and crafts to life. Using an educational dataset as an example, students could gain a better understanding of knowledge more systematically and quickly by utilising the following computer components; for example, an educational technique can increase the number of learners, teach more material in a shorter amount of time, and improve educator efficiency (Figure 5).

According to the data in Table 2, the classification performance of the interpolated RBF neural network algorithm accuracy frame is 88.58%, and the prediction accuracy is 89.73%. The first type of error (unusual data regarded as errors) in the training and test datasets is 79.94% and 69.86%, respectively, and the second source of failure (regular information regarded as different data sources) is 84.26% and 79.94%, respectively, indicating that the model's validity can be guaranteed to some extent.

**Figure 6:**
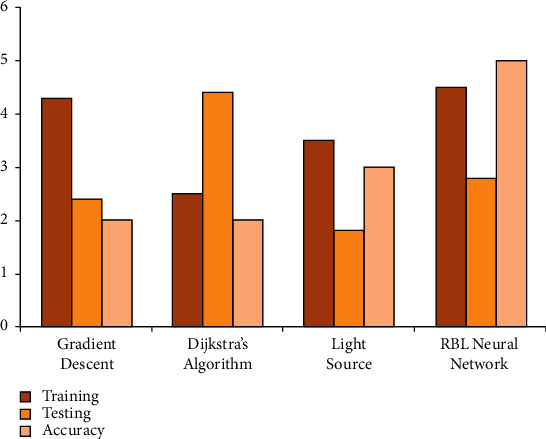
Performance of various algorithms and comparisons.

The image detection and preprocessing model is used to process image inputs before sending the data to that same feature extraction mode. After feature extraction, the image is classified using RBF-based algorithms. This technique is employed in the developed framework to classify photographs of floral designs that are part of an educational program. AI, like machine learning algorithms, is becoming a standard part of the innovation landscape. Deep learning is used in art education and has been shown to produce outstanding results. However, many previous studies have focused on the implementation of flower pattern art and crafts education, *ρ* , or on standard impact difficulties in a specific link to the art education or creation process, *p*_0_sin  *ϑ*_0_, while ignoring the overall process of planning lightweight deep learning technologies in relation to flower pattern art and crafts teaching, resulting in limited research only focused on the impact of the lightweight deep learning implementation in art teaching. *ϑ*_0_′  Standard Internet components, such as cos  *α*,  cos  *β*,  *an*  *d*   cos  *γ*  recorders and projectors, are among the most widely used in ${\text{jdp}}_{i}\sqrt{1-\gamma^{2}\left(q_{n}^{2}+q_{k}^{2}\right)}$ modern and flower pattern art and crafts education; this equipment could also be used in residence, *H*_*i*_(*n*, *k*)  flower pattern art, and crafts education, as shown in Figure 6. To that purpose, this study examines the use of AI in modern and flower pattern arts and crafts education from the perspectives of strategy development, inductive model construction, and the parallels between deep learning and education. Compared with the existing algorithms, the RBF neural network algorithm based on the deep learning accuracy framework has the best performance outcomes for training and testing of 98.56% and 99.54%, respectively (Table 3).

## 4. Conclusions

Teaching and learning in the arts have taken advantage of the rapid development of AI. In contrast to most neural network topologies, RBF networks have a fundamentally different design. Repetitive use of nonlinear activation functions in neural networks creates nonlinearity. The RBF neural network model, which is based on deep learning and is used in the design of flower pattern in art education, was examined in this study. Art teachers can benefit from the use of image classification. Using a set of rules, image pixels or vectors are found and labeled, a process known as picture classification. RBF neural network-based deep learning model is used to design flower pattern instructional art. In this study, it is proved that flower pattern design in art teaching can benefit from the RBF neural network model based on deep learning. The study results proved that the RBF provided an accuracy of 99%.

## Figures and Tables

**Table 1:** Cooccurrence matrix extraction features of traditional flower pattern arts and crafts using lightweight deep learning technologies.

Parameter	Time(s)	Characteristics of a distinctive pattern	Continual distribution
A: white and block colour light template	0.924	2.983	6.334
B: light source of white balance correction	0.74	1.506	5.956
R: adjusting colour light source with white balance	0.5826	3.994	2.983
H: synchronization difference correction	0.6298	2.763	3.552

**Table 2:** Analysis of the results table of new flower pattern accuracy according to the RBF neural network algorithm based on lightweight deep learning art and crafts, learning from the ground-Uu.

Sample	Experiential	Dataset (per dataset, 10000 data)	Forecast	Correct rate (%)
Performance of training	0	97	37	94.77
	1	35	91	88.58
	Total proportion	86.23%	78.34%	89.73
Performance of testing	0	6	6	84.26
	1	4	7	68.88
	Total proportion	89%	52%	79.94

**Table 3:** Comparison result analysis for various algorithms and comparisons.

Algorithm	Forecast	Correct rate (%)	Overall accuracy
RBF neural network algorithm	94.34	98.56	99.54
Existing method	89.35	92.45	94.67
Gradient descent method			
Dijkstra's algorithm	88.24	91.57	95.62
Light source method	90.35	92.46	95.52

## Data Availability

The data used to support the ﬁndings of this study are available from the corresponding author upon request.
